# The plant terpenoid carvone is a chemotaxis repellent for *C. elegans*

**DOI:** 10.17912/micropub.biology.000231

**Published:** 2020-03-13

**Authors:** Clayton T. Ellington, Andrew J. Hayden, Zack B. LaGrange, Marina D. Luccioni, Mohammed A.M. Osman, Lauren I.E. Ramlan, Miranda A. Vogt, Sujay Guha, Miriam B. Goodman, Lauren A. O'Connell

**Affiliations:** 1 Organismal Biology Lab BIO161, Stanford University, Stanford, CA 94305; 2 Department of Molecular and Cellular Physiology, Stanford University, Stanford, CA 94305; 3 Department of Biology, Stanford University, Stanford, CA 94305

**Figure 1. C. elegans show negative chemotaxis behavior in response to the plant terpenoid carvone:**
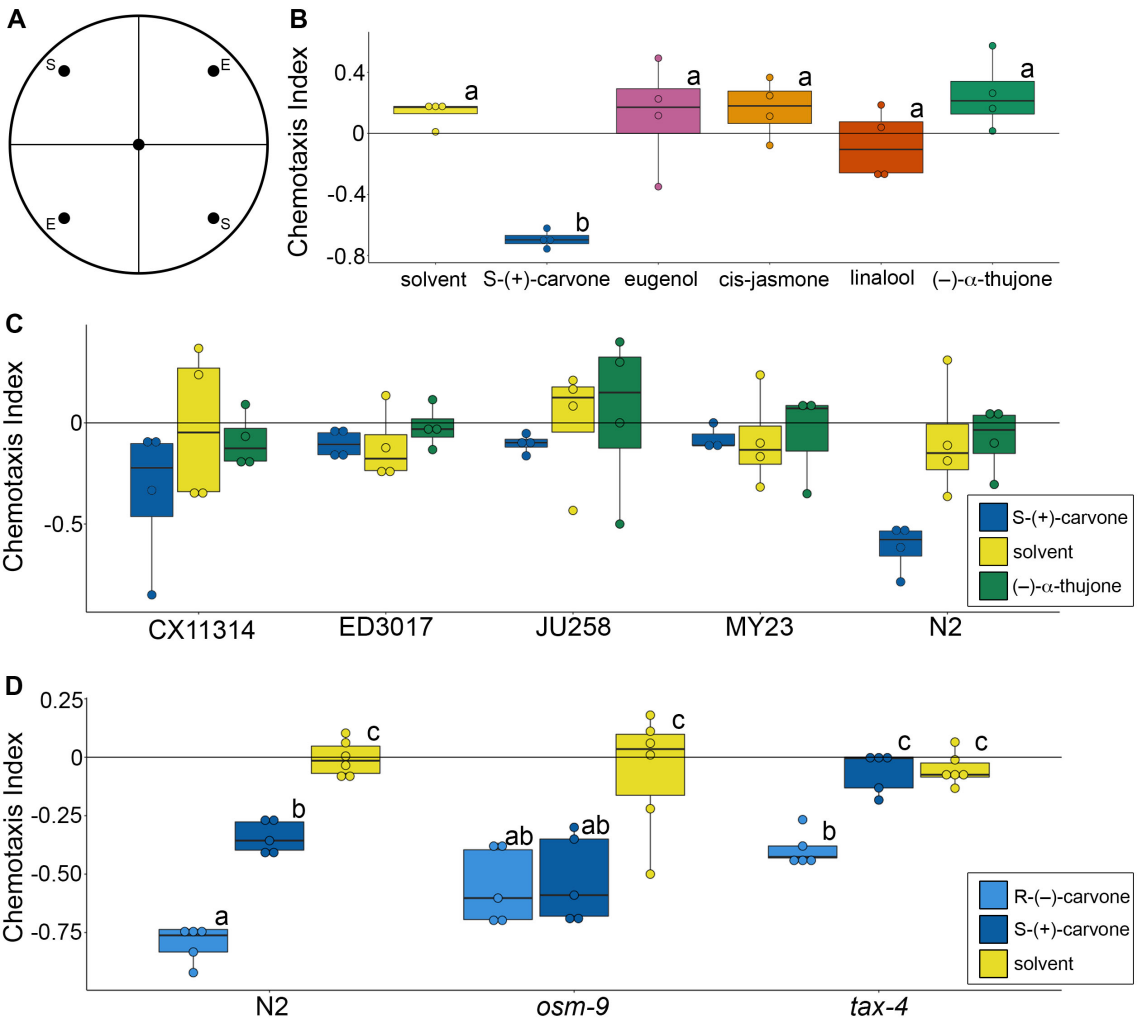
**(A)** Chemotaxis assays were performed on 35 mm circular plates divided into quadrants. Behavior in the N2 wild type strain was scored in response to experimental compounds (E, plant compounds dissolved in ethanol) and solvent (vehicle control, 100% ethanol). **(B)** Chemotaxis behavior was neutral for solvent and most plant compounds, including eugenol (pink), cis-jasmone (light orange), linalool (dark orange), and (–)-ɑ-thujone (green). Worms were repulsed by S-(+)-carvone (dark blue). Groups not connected by the same letter are significantly different. **(C)** Chemotaxis behavior was scored across divergent worm strains in response to S-(+)-carvone (dark blue), solvent (yellow) and (–)-ɑ-thujone (green). There was a significant effect of compound, but not strain, on chemotaxis response. **(D)** Chemotaxis behavior in response to stereoisomers of carvone, including S-(+)-carvone (dark blue) and R-(–)-carvone (light blue), was measured in wild type (N2) and two chemosensation mutant strains, *osm-9* and *tax-4*. Groups not connected by the same letter are significantly different.

## Description

Plants synthesize many structurally diverse organic molecules to interact with the organisms around them. These compounds can act at the receptor level in target organisms to aid the plant in defense or to facilitate a symbiotic relationship (Bahar *et al.*, 2007; Wink, 2018). We have not exhausted these plant-derived secondary metabolites as a source of novel drug therapeutics for pressing human health challenges because screening them for potential function as neuronal actuators is difficult. Here, we explore (1) using *C. elegans* as an efficient and practical organism for screening plant compounds for potential bioactivity in the nervous system, and (2) using these methods in an undergraduate classroom to bring real scientific discoveries to teaching laboratories. *C. elegans* are well studied, have essential genetic tools, short generation times, and easy culture techniques (Corsi *et al.*, 2015; O’Reilly *et al.*, 2014). Moreover, *C. elegans* navigates its environment by relying on chemosensation, triggering chemotaxis in response to odorants (Bargmann, 2006; Margie *et al.*, 2013). These characteristics make it a potentially effective organism for screening phytochemicals and teaching in an undergraduate laboratory setting.

We first screened five commercially available plant compounds for potential actuators of neuronal function using *C. elegans* chemotaxis behavior (Figure 1A), including (S)-(+)-carvone, eugenol, cis-jasmone, linalool, and (–)-ɑ-thujone (Figure 1B). The chemotaxis response in the N2 wild type strain differed across plant compounds and solvent control (ANOVA, F(5)=10.52, p=7.59×10^-5^). In particular, (S)-(+)-carvone elicited a strong repulsion effect compared to solvent (p=0.0004) and all other compounds ((S)-(+)-carvone versus: eugenol p=0.005, cis-jasmone p=0.003, linalool p=0.008, (–)-ɑ-thujone p=0.00009). No other compounds significantly differed between each other or solvent control at the concentration that was tested (20 mM), which is surprising as these compounds have been previously shown to produce interesting neuromodulatory and behavioral effects in other animals: cis-jasmone repels aphids (Birkett *et al.*, 2000), (–)-ɑ-thujone is a GABA_A_ receptor antagonist (Höld *et al.*, 2000), and both linalool (Batista *et al.*, 2008) and eugenol (Chung *et al.*, 2014) alleviate glutamate-induced nociceptive pain. It may be that dose-response curves or different genetic strains would be required to fully explore the effects of these compounds in nematodes. Regardless, to our knowledge, this is the first demonstration of carvone as a repellent in *C. elegans* and we conducted follow-up experiments to gain insight into potential mechanisms.

We next tested whether there is genetic variation in chemotaxis response elicited by plant compounds by comparing chemotaxis behavior across divergent *C. elegans* strains (Figure 1C). Such variation between strains could allow for genome wide association studies to identify potential neuronal substrates (Cook *et al.*, 2017). We tested (S)-(+)-carvone as well as (–)-ɑ-thujone due to its known interactions with GABA_A_ receptors (Olsen, 2000). Across four wild isolate strains and the N2 wild type strain, there was a significant effect of compound, but not strain, on chemotaxis behavior (2-way ANOVA, Compound: F(2)=4.679, p=0.0136; Strain: F(4)=1.821, p=0.1392). (S)-(+)-carvone elicited a negative chemotaxis response compared to both solvent (p=0.0409) and (–)-ɑ-thujone (p=0.0201). The chemotaxis behavior elicited by (–)-ɑ-thujone did not significantly differ from solvent (p=0.9444), mirroring results from the first screening experiment. These findings indicate that screening the number of strains and replicates required for genome wide association mapping is outside the scope of a single small undergraduate laboratory course, but leaves open the possibility that screening additional wild isolates could be valuable.

To explore the potential neuronal mechanisms for the repellant effects of carvone, we screened worm strains with mutations in chemosensory signaling pathways (Figure 1D). We used *tax-4* mutants, which lack the alpha subunit of a cyclic nucleotide-gated ion channel required for chemosensation (Komatsu *et al.*, 1996), and *osm-9* mutants, that lack a transient receptor potential (TRP) channel subunit that is also needed for chemosensation (Bargmann, 2006; Colbert *et al.*, 1997). The *tax-4* and *osm-9* channels are expressed in non-overlapping subsets of sensory neurons. In addition to using mutant strains, we also examined both stereoisomers of carvone, (R)-(–)-carvone and (S)-(+)-carvone, which are recognized as different odors by humans and thus may bind different olfactory receptors (Leitereg *et al.*, 1971). Both worm strain, compound, and their interaction had a significant effect on chemotaxis behavior (2-way ANOVA, Strain: F(2)=11.085, p=0.0002; Compound: F(2)=64.929, p=3.88×10^-13^; Strain * Compound: F(4)=7.476, p=0.0001). The two stereoisomers of carvone were more repellant than solvent control (solvent vs. (R)-(–)-carvone, p<0.0001; solvent vs. (S)-(+)-carvone, p=4.0×10^-6^) and (R)-(–)-carvone seemed to elicit stronger responses than (S)-(+)-carvone (p=9.2×10^-6^). Chemotaxis behavior in the *tax-4* strain was significantly different than both *osm-9* (p=0.0006) and N2 wild type strains (p=0.0006). We did not detect any differences in responses to carvone between *osm-9* and N2 (p=0.9998) in this assay. Given the variance in these assays, however, more replicates will be needed to fully elucidate the role of *osm-9* in carvone chemosensation and the apparent difference in sensitivity to carvone stereoisomers.

In summary, we have shown here that the terpenoid carvone, derived from caraway, dill and spearmint, has a repellent chemotaxis effect in *C. elegans*. Our results support other studies showing carvone is an effective insect repellent (Franzios *et al.*, 1997; Tripathi *et al.*, 2003). The repulsion effect of the carvone enantiomers was reduced in *tax-4* mutants, suggesting that both enantiomers are sensed by *tax-4*-expressing sensory neurons. Overall, this work shows that carvone is a *C. elegans* repellant and also establishes the feasibility of screening for bioactive plant compounds in a classroom undergraduate research experience (CURE) course. This approach allows students to pursue genuine scientific discoveries within a few classroom sessions.

## Methods

Worm strains and chemicals

Strains were obtained from the Caenorhabditis Genetics Center (CGC) at the University of Minnesota and CeNDR at Northwestern University (Reagents). Animals were maintained in 20°C incubators. Synchronized nematode cultures were initiated by bleaching adults to obtain eggs. Collected eggs were seeded on 10 cm Nematode Growth Media plates (approximately 300 eggs per plate), spread with OP50 *E. coli*. Eggs were allowed to hatch on these plates and kept at 20 °C for approximately 65 hours when the population reached the young adult stage. These young adult worms were used for chemotaxis assays (Stiernagle, 2006). The wild strains are part of the recommended starter pack “divergent set” from CeNDR; strains used in the experiments described were chosen for testing because the nematodes did not dig into or crawl out of the growth or chemotaxis plates.

We used commercially available phytochemicals in this study, including cis-jasmone (TCI chemicals, Cat # J0003), eugenol (TCI chemicals, Cat # A0232), linalool (TCI chemicals, Cat # L0048), (R)-(–)-carvone (Sigma-Aldrich, Cat # 124931), (S)-(+)-carvone (Sigma-Aldrich, Cat # 435759), and (–)-ɑ-thujone (TCI chemicals, Cat # T0989). All compounds were dissolved in 100% ethanol and were used at 20 mM final concentration.

Chemotaxis assays

Chemotaxis assays were run by undergraduate students in a laboratory course. Students performed the assays blind to compound and *C. elegans* genotype until all data had been submitted to the instructor. Chemotaxis plates [5mM KPO_4_ (pH 6), 1mM CaCl_2_, 1mM MgSO_4_, 2% agar] were divided into four quadrants with a dot for placing experimental (E) or solvent (S) compounds (Figure 1A). On each plate, 5uL of experimental compound or solvent was placed on each dot and the plate was incubated for 30 min to form a chemical gradient. During this time, worms were removed from plates and washed three times with Chemotaxis Assay Buffer [5mM KPO_4_ (pH 6), 1mM CaCl_2_, 1mM MgSO_4_]. Roughly 100 worms were transferred into the center of each plate. Then, 2 uL of a 1% sodium azide solution was placed on the compound dot for each quadrant to immobilize worms at these locations. After application of the paralytic, the excess liquid from the worm transfer was removed with a KimWipe under a dissection microscope, allowing the worms to roam the plate for either 1 hour (experiment 1 with multiple plant compounds) or 30 min (all other assays). Worms were counted manually by students using a dissection microscope and a tally counter. If a worm was still in the center dot or overlapping quadrants, it was added to the total number of worms but not counted for any quadrant’s count. Seven students conducted all the experiments described (one figure panel was done in one laboratory session), where each test was replicated 4-6 times with roughly 100 worms on each plate. If a plate had fewer than 20 worms total, it was removed from data analysis. The chemotaxis index (CI) was calculated for each experimental plate, where positive values suggest attraction and negative values suggest repulsion: CI = (Number of worms in the two experimental quadrants – Number of worms in the two solvent quadrants) / Total number of worms on the entire plate.

Data analysis

All data analysis and figure generation was performed in RStudio (R version 3.5.2). For all experiments, one- or two-way ANOVAs (aov function in R) were used to determine significant differences between groups using the chemotaxis index as the dependent variable and compound and/or worm strain as independent variables. Tukey HSD was used for post-hoc comparisons adjusted for multiple comparisons. Assumptions of parametric tests were met by all datasets, which was confirmed using Levene’s test (leveneTest in R) to confirm homogeneity of variance and the Shapiro-Wilk test (shapiro.test in R) to confirm normality of residuals.

Classroom pedagogy

We conducted the experiments described here across three 4-hour laboratory sessions. As this was a pilot course, there were seven students enrolled. These laboratory sessions with worm chemotaxis experiments were proceeded with an initial training session where students learned how to conduct chemotaxis assays using a known attractant (isoamyl alcohol) and repellant (2-nonanone). Weekly homework included reading relevant literature (Cook *et al.*, 2017), data visualization, statistical analysis in R, and writing the results and interpretations. The final project was to write this journal style article.

## Reagents

**Table:** 

**Strain Name**	**Genotype**	**Source**
N2 (Bristol)	Wild type	Caenorhabditis Genetics Center (CGC) at the University of Minnesota
CX11314, ED3017, JU258, MY23	Wild isolates	CeNDR at Northwestern University
CX10	*osm-9(ky10)* IV	Caenorhabditis Genetics Center (CGC) at the University of Minnesota
PR678	*tax-4(p678)* III	Caenorhabditis Genetics Center (CGC) at the University of Minnesota
